# Housing and Health: Here We Go Again

**DOI:** 10.3390/ijerph182212060

**Published:** 2021-11-17

**Authors:** Lorenzo Capasso, Daniela D’Alessandro

**Affiliations:** 1 Italian Ministry of Education, USR Abruzzo (Regional Office of Abruzzi), 66100 Chieti, Italy; 2 Department of Civil Building and Environmental Engineering, “Sapienza” University of Rome, 00100 Rome, Italy; daniela.dalessandro@uniroma1.it

**Keywords:** housing and health, built environment, indoor health, public health

## Abstract

Housing is one of the major determinants of human health and the current COVID-19 pandemic has highlighted its relevance. The authors summarize the main issues, including dimensional standards, indoor air quality, safety, accessibility, neighborhoods, and area characteristics. The authors propose an operating scheme in order to implement actions to improve residential wellbeing on a local, national, and international level.

## 1. Introduction

Our readers might wonder, why another special issue on housing and health? Is it really necessary to study and work on the relationships between housing and human health? Is it still a relevant issue in today’s world? We are quite sure that the answer is yes, because housing was, is, and will remain a central theme for public health worldwide. We must strengthen our knowledge to ensure the best and healthiest living condition for the entire world population. We therefore strongly need new research on this topic, not just to evaluate the current situation in different countries and contexts, but also to be able to validate new solutions aimed at improving the health standards of housing and making them suitable and affordable for a larger portion of the human population.

## 2. Relevance of Housing for Human Health

Housing represents a core and traditional topic of public health [1], despite having been wrongly considered an “old” topic not worthy of attention by many public health researchers and practitioners in different countries [2]. International studies widely and strongly recognize the indoor environment as a major health determinant [1,3,4,5,6], in both developing and developed countries [7,8,9]. In the most economically developed countries, nowadays, people spend up to 90% of their lifetime indoors [6,10,11], while in developing countries, considerable levels of indoor pollution make housing unsafe, with a remarkable impact on the health of inhabitants [9,12,13]. The occurrence and re-occurrence of pathologies related to the quality of built environments, exacerbated by the severe current socio-economic crisis, upholds once more the ultimate importance of the domestic environment as a principal living space [6,9,11,13,14]. The current pandemic conditions have exacerbated housing, social, and sanitary problems, highlighting different needs in different countries and contexts [15,16]. Several issues characterize dwellings’ impact on human health, but considering demographical, socioeconomic, and epidemiological factors, we might summarize the main housing issues, that in our opinion ought to be explored, as these key problems:

- Dimensional standards
- Indoor air quality
- Safety
- Accessibility
- Neighborhoods and area characteristics

Relevant classical issues such as thermal and hygrometric comfort, lighting, noise protection, water supply, and waste disposal have a strong impact on both psychological and physical health of inhabitants. Despite being widely recognized as key factors for healthy living environments at both scientific and legislative levels, these factors are not adequately guaranteed in all dwellings worldwide [5,6,17,18,19]. New emerging threats and opportunities for indoor health have emerged in recent years and must be investigated, among which are surely electromagnetic fields and privacy, linked to new technologies and domotics [20,21,22,23,24,25,26,27,28,29,30].

## 3. Dimensional Standards

Sufficient dimensional area is a key determinant for healthy housing [31]. According to the World Health Organization (WHO), in terms of living space, dwellings must be large enough to comfortably accommodate people of different ages and must guarantee sufficient space to fulfill the safety and privacy needs of the occupants [32,33]. Dwellings nowadays ought not only to meet a basic human need for shelter, but also contribute to the physical and psychological well-being and social integration of the residents [33,34]. Housing with substandard spacing is linked with mental and infectious diseases [4,6,33,35]. The strong correlation between the built environment, overcrowding, and the spread of infectious diseases is well documented in particular for some pathologies such as tuberculosis and epidemic cerebrospinal meningitis, and is now coming back as a fundamental threat due to the COVID-19 pandemic [6,33,36,37,38,39,40,41]. Insufficient housing space conditions and the lack of privacy can be related to psychiatric conditions such as anxiety and depression [32,42,43]. At the same time, it represents an issue for people with physical disabilities, particularly if they require mechanical supports [17,32]. Space shaping can also influence social interaction and integration, determining how much interaction occurs and its results [19,32,33]. Defining the appropriate amount of internal space for a dwelling is a complex issue and there is not a vast amount of evidence-based data on space standards. It is not only related to floor area, but also to several different parameters that can influence air circulation and space perception, among which are ceiling heights, walls colors, rooms proportions (length, depth, diagonals), windows size, and natural lightening [6,44,45,46].

## 4. Indoor Air Quality

Indoor air is an historical and widely recognized health determinant [3,4,5,6,7,47,48,49,50]. The first identified pollutant was carbon dioxide, discovered by Pettenkofer in the second part of the 19th century [51]. Subsequently, the number of new pollutants has been widened, and many actions have been taken to improve indoor air quality [7,50,51,52,53,54]. Low-quality indoor air is recognized as a risk factor for chronic and acute diseases such as neoplasms, asthma, respiratory infections, eczema, and rhinitis [55,56,57,58,59,60,61,62]. Important public health goals were achieved by ameliorating building ventilation [6,7,39,51,53,63,64,65,66,67,68,69] and tackling tobacco smoke, radon, formaldehyde, and other volatile organic compounds (VOCs) [6,7,66,67,70,71,72,73,74,75]. The identification of sick building syndrome, building-related illnesses, and multiple chemical sensitivity provided an important base for further indoor air improvements [76,77,78,79,80,81]. A cause of concern, at least in the most economically developed countries, is the decrease of air exchange rates related to energy saving technologies and policies, which may generate a future deterioration of indoor air quality [82,83,84] In developing countries, indoor air quality is often still extremely poor, and a major health threat is represented by the combustion of solid fuels and, in general, by household cooking and space heating [8,12,85,86].

## 5. Safety

Despite a lack of reliable epidemiological data on a global basis, home accidents are among the leading causes of death, infirmity, and disability worldwide, in both developing and developed countries [87,88,89]. The most common accidents include falls, poisoning, fire and burning, choking and suffocation, drowning, and submersion [90,91]. Particularly fragile categories are children and the elderly [86,92,93,94,95]; among the elderly, accidental falls cause worldwide large mortality and even larger morbidity and disability [95,96], representing a rising public health issue because of the population aging [97,98,99,100,101].

Among emerging safety issues, we would also include structural problems related to extreme natural phenomena, such as floods, tsunamis, earthquakes, landslides, volcanic activities, and heat and cold waves. In the last few years, several catastrophic events have occurred around the globe, causing many victims [17,102,103]. Dwellings should provide a secure shelter from these threats and not cause further harm. Intense land use, climate changes, urbanization, poor building quality, and the spread of informal settlements have increased the frequency and intensity of these tragedies [104,105]. These events can generate a deterioration of indoor environmental quality even when buildings resist the impact, worsening previously poor living conditions, as may happen in the case of shacks, basements, semi-basements, and garrets [104,105,106,107].

## 6. Accessibility

The aging population faces increasing morbidity, multi-morbidity, functional limitations, and disability [108,109]. Although aging is not a disease *per se*, the elderly are more fragile and susceptible to the harmful effects of their dwellings; therefore healthy ageing is a goal for an economically and socially sustainable future [17,110,111,112,113,114,115]. Of course, not only the elderly have movement limitations that need dwelling adaptation; such adaptation is also needed for many temporary, chronic, and acute conditions, among which are injuries from road, home, or work accidents, and genetic, neurodegenerative, autoimmune, cardiovascular, and infectious diseases [116,117]. The chance of living a healthy life for old and disabled people is linked to the possibility of using suitable indoor and outdoor spaces, especially at home, to conduct an independent and possibly active life [118,119,120]. To ensure these possibilities, living spaces ought to be designed for people with functional limitations, guaranteeing at least the activities of daily living (ADLs): bathing, dressing, ambulation, toileting, and feeding [119,120,121]. Accessibility for disabled people and safety for fragile people should be merged in new adapted/adaptable housing design.

## 7. Neighborhoods and Area Characteristics

As is already widely expressed in international literature, the concept of healthy housing also implies a residential setting that can fulfill the expectations of its residents [71,122]. In fact, the area characteristics include a broad set of aspects of both the physical and social environment, which exerts a powerful influence on health opportunities for the population [123,124,125,126,127,128,129,130,131]. For example, an urban morphology with high density seems to facilitate healthier choices, at least in terms of attitude towards physical activity, than urban forms characterized by scattered settlements and low residential density [132]. The presence of land-use mix, frequent road intersections between residential and commercial areas, etc., increase walkability, thereby facilitating direct pedestrian paths between various destinations [132]. At the same time, density can increase pollution, if neighborhoods are not well designed or managed. Air quality, noise, water supply, municipal waste management, transportation, and green areas represent features of the built environment that directly and indirectly impact citizens’ health [71,122]. If the availability of environmental and social services differs among neighborhoods, the population’s opportunities (including those for health) change, determining strong inequalities across and within cities [133,134]. For example, in peripheral areas, where low-income communities generally live, it is easier to find urban voids, abandoned buildings, and degraded lots, all conditions related to segregation and to an increased risk of violent behaviors [134]. Fear of crime is one of the most significant social problems in cities, negatively influencing people’s lifestyle and mental health [38,135,136,137,138]. These problems are mainly observed in badly maintained housing estates with large housing blocks, which are poorly lit and receive little maintenance, along with large public open spaces with unclear management responsibilities [71,122].

## 8. Perspectives

As housing is a leading health determinant, improving living conditions in both economically developed and developing countries may reduce mortality and burden of disease, and increase health quality from a physical, psychological, and social point of view [3,5,6,17,49,50,139]. This goal can be easily combined with other socioeconomic challenges, such as poverty reduction, mitigation of climate changes, soil protection, and improving social cohesion, in accordance with several sustainable development goals (SDGs) of the United Nations Foundation 2030 Agenda (such as number 3, 6, 10, 11, 13, and 15) [17,140]. So far, we have briefly summarized some priorities in the field of housing and health; to achieve things at the local, national, and international levels, our proposals to be implemented are:

- **R**esearch, to consolidate evidence on the main health threats in indoor environments and to create strategies to mitigate their effects, as well as elaborating on guidelines and standards;
- **E**ducation, to spread research-acquired knowledge not only to health sector workers, but to engineers and architects, lawmakers, public administrators, and the general population (directly or via relevant stakeholders);
- **L**awmaking, to enforce research-based regulation, as it is recognized as key part of health protection in built environments [9,46,69,141,142,143];
- **P**olicy, to provide political and financial support to housing in local, national and international programs;
- **E**valuation and control, to obtain a constant monitoring of implemented housing campaigns in input, output, and process validated indicators, and control of local health authorities on hygienic standards application.

To successfully implement these proposals, which we may call RELPE actions, strong commitment and functional coordination and harmonization is needed at a local, regional, national, and international level, providing the involvement of researchers, educators, public health operators, building designers, urban planners, lawmakers, communities, and representative associations.

## 9. Conclusions

Housing is a key determinant for human health. We have tried to summarize some of the major factors connecting housing and health, citing the most relevant scientific evidence currently available, that shows a strong relation with social and economic conditions; however, it is often very difficult to assess their independent effect [144]. The COVID-19 pandemic has strongly highlighted housing issues worldwide, especially for the lower socioeconomic part of different societies [41]. “Housing and health” as a subject should be approached with a multidisciplinary and transdisciplinary strategy in both research and practice [145,146,147] because of the complexity and wideness of its related aspects. In the last years, several studies have demonstrated the large potential for better human health through improvement of the living environment [58,148,149]. These results offer useful indications for the development of good practices to ameliorate built environment characteristics and make them available for all involved actors [17,71]. At the same time, a clear and updated regulatory system is a critical key factor to ensuring public health protection, relying on the most recent acquisitions of international scientific literature [9,14,47,70,150]. Innovative strategies that can match together different aspects (technical building, urban, social, legal, political), such as the RELPE that we have modestly suggested, should be elaborated, discussed, experimented, evaluated, and implemented, to achieve things on the local, national and international levels, in order to achieve the desired results in terms of public health.
